# Recombinase polymerase amplification combined with lateral flow dipstick assay for rapid visual detection of *A.simplex (s. s.)* and *A.pegreffii* in sea foods

**DOI:** 10.1016/j.heliyon.2024.e28943

**Published:** 2024-04-07

**Authors:** Xiaoming Wang, Ting Xu, Siling Ding, Ye Xu, Xingsheng Jin, Feng Guan

**Affiliations:** a College of Life Sciences, China Jiliang University, Hangzhou 310018, China; b Zhejiang Museum of Natural History, Hangzhou 310018, China

**Keywords:** *A. simplex (s. s.)* and *A. pegreffii*, Recombinase polymerase ampliﬁcation, Lateral ﬂow dipstick, Visual detection

## Abstract

Anisakiasis is a food-borne parasitic disease mainly caused by the third stage of *Anisakis simplex (s. s.)* and *Anisakis pegreffii.* Traditional methods for detecting of *Anisakis* involve morphology identification such as visual inspection, enzyme digestion, and molecular methods based on PCR, but they have certain limitations. In this study, the internal transcribed spacer 1 (ITS 1) regions of *Anisakis* were targeted to develop a visual screening method for detecting *A. simplex (s. s.)* and *A. pegreffii* in fish meat based on recombinase polymerase amplification (RPA) combined with lateral flow dipstick (LFD). Specific primers and probes were designed and optimized for temperature, reaction time, and detection threshold. LFD produced clear visual results that were easily identifiable after a consistent incubation of 10–20 min at 37 °C. The whole process of DNA amplification by RPA and readout by LFD did not exceed 30 min. In addition, the detection limit is up to 9.5 × 10^−4^ ng/μL, and the detection of the artificially contaminated samples showed that the developed assay can effectively and specifically detect *A. simplex (s. s.)* and *A. pegreffii*, which fully meet the market's requirements for fish food safety supervision.

## Highlights

• An infect rate investigation and molecular identification of *Anisakis* in two marine fish in the East China Sea was conducted.
• A new detection method for *Anisakis simplex (s. s)* and *Anisakis pegreffii* detection was developed.
• The developed method does not require complicated instruments, and it can be completed within 30 min.
• A new detection method can detect pathogenic *Anisakis* in fish meat.

## 1 Introduction

*Anisakis* is an important food-borne zoonotic parasite prevalent worldwide, and humans are infected by consuming raw or undercooked fish, including hairtail, small yellow croaker, mackerel, sturgeon and threadfin bream, but also red sea bream, black seabream, red drum larvae and other species [1,2]. And the infection rate in some fish samples can reach 80 % [[3], [4], [5], [6], [7]]. When ingested, according to the different infect sites, *Anisakis* can lead to one or more symptoms, such as eosinophilic granulomas and intestinal ectopic phenomena [8,9] and lead to allergic symptoms [[10], [11], [12]], vomiting, diarrhea, and pleural effusions [13] and anisakisasis, which was first observed in the Netherlands in 1960 [14]. Anisakiasis is principally caused by the consumption of raw or undercooked fish products containing living third-stage *Anisakis* larvae (L3) [[15], [16], [17]]. Now, Anisakiasis has become an increasing human health concern in the world. The Anisakiasis cases had been reported in 34 countries, it is not only reported in Asian regions such as Japan, South Korea and China, but also frequently appears in European regions such as Italy, Spain, and France [17,18]. Thus, as the European Food Safety Authority released a scientific opinion on control of parasite of fishery products, it is necessary to develop more accurate detecting methods to supervise causative agents of Anisakiasis in sea food [19].

Several techniques have been developed and used to identify *Anisakis* in food safety supervision and clinical examination. Traditional identification methods involve morphology identification, such as visual inspection, enzyme digestion, and ultraviolet-press methods [[20], [21], [22]]. Morphological identification is simple, rapid, and practicable; yet, damage or fragmentation during the egg stage or early stages of infection in fish and humans [23,24] and variations in structure and morphology during larval different developmental stages of *Anisakis,* as well as the impact of external environmental factors are some of the main challenges using this method for *Anisakis* detection in processed products such as surimi and canned fish [25].

The development of biotechnology has driven the progress of a variety of DNA detection techniques for species identification and has become an important candidate for parasite species identification. For example, PCR [26], PCR-RFLP [27], and real time-PCR (qPCR) [[28], [29], [30], [31]] also had been widely used in this detection field. These methods have satisfactory sensitivity and specificity. However, common PCR-based detection is relatively complex and time-consuming. To circumvent the limitations of PCR-based methods, some researchers have developed the isothermal method. For example, the loop-mediated isothermal amplification (LAMP) assay and recombinase polymerase amplification (RPA), these isothermal methods are considered rapid and simple assays [32].

RPA technology exhibits significant potential for nucleic acid detection owing to its simplicity, high sensitivity, and ability to react at low and consistent temperatures without relying on complex instrumentation [[33], [34], [35]]. The RPA reaction system is typically sufficient for 10–20 min detection at 37 °C. RPA has been widely used for detecting bacteria [36,37], fungi [38,39], viruses [40,41], and parasites [42,43], with the aid of fluorescently labeled probes or the lateral flow dipstick (LFD) for visual analysis of the amplified products. Additionally, the RPA reaction system has advantages in identifying specific genes, e.g., those linked with drug-resistant genes, transfected genes, and novel coronaviruses-specific genes [7,35,44]. Consequently, this innovative assay is a significant technique for development in the detection field.

In this study, the internal transcribed spacer 1 (ITS 1) regions of *Anisakis* were targeted to develop a visual screening method for the main pathogen of *Anisakis* detection in fish meat based on RPA combined with LFD. The aim is to provide technical support for the onsite identification of *Anisakis* to ensuring food safety and preventing Anisakiasis.

## 2 Materials and methods

### 2.1 Samples and DNA extraction

The nematodes utilized in this study were sourced from 54 small yellow croaker *(Larimichthys polyactis)*and 13 mackerel (*Pneumatophorus japonicus*) specimens which were retrieved from the East China Sea in August 2022, and the sampling location listed in Fig. S1. Following fish dissection and observation, all the nematodes were collected under a stereo light microscope (SLM), then washed with saline solution (pH = 7.4) and fixed in 70 % ethanol solution. Subsequently, the number of nematodes was recorded and analyzed, and the statistics were listed in Table S1. Randomly selected three nematode individuals from each host and then were washed using distilled water, and genomic DNA was extracted using an Animal Tissue Genomic DNA Extraction Kit (Hangzhou Xinjing Biotechnology Co., Ltd.). Ten identified known *A. simplex (s. s*.) nematode bodies used as positive control were obtained from the Marine Fisheries Research Institute of Zhejiang Province, Zhoushan. The DNA purity and concentrations were evaluated using NanoDrop 2000 (ThermoFisher, USA).

### 2.2 Species identification for nematodes

The species for Anisakidae nematodes were further identified using the PCR-RFLP method following a previously described approach [27]. A forward primer NC5: 5′-GTAGGTGAACCTGCGGAAGGATCATT-3′ and reverse primer NC2: 5′-TTAGTTTCTTTTCCTCCGCT-3′ were used to amplify the ITS 1,*5.8S* ribosomal RNA gene, and internal transcribed spacer 2 (ITS 2) regions, which were digested with the *Hinf*I enzyme. The primers were synthesized by Tsingke Biotechnology Co., Ltd. (Hangzhou, Zhejiang). The digestion products were analyzed on 2 % agarose gel. Simultaneously, the determinate samples were dispatched to Hangzhou Tsingke Biotechnology Co., Ltd. for DNA Sanger sequencing. The sequencing results were compared and assembled using Clustal X software (version 2.1) and further Blast in GenBank database.

### 2.3 RPA primer design and amplification test

**Fig. 1:**
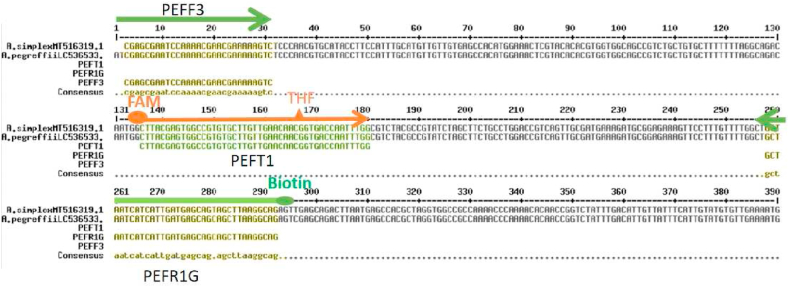
Location of primers and probe in ITS 1 sequence. PEFF3 represents the forward primer, PEFR1G represents the reverse primer, and PEFT1 represents the nfo probe.

The ITS sequences of *Anisakis* were also selected as the target gene to develop an RPA-LFD assay. Additionally, ITS 1 sequences of nine nematode species, including *A. simplex (s. s.)* (No. MT516319.1), *A. pegreffii* (No. LC536533.2), *Anisakis typica* (No. FJ161072.1), *A. physeteris* (No. KY826440.1), *A brevispiculata* (No. KY352231.1), *Contracaecum* (No. KF990496.1), *Gnathostoma* (No. JN408329.1), *Raphidascarisand* (No. MW371020.1) and *Hysterothylacium* (No. HQ702733), were downloaded and compared to search specific sequences. The primers and probe were designed using Clone Manager software, and the sequences are listed in Table 1; their positions are shown in Fig. 1.

**Table 1:** RPA primers and probe sequences of *Anisakis*.

Primer/Probe	Sequence(5′-3′)	Product size(bp)
PEFF3	CGAGCGAATCCAAAACGAACGAAAAAGTC	291
PEFR1G	(Biotin)-CTGCCTTAAGCTGCTGCTCATCAATGATGATTAGC	
PEFT1	(FAM)-CTTACGAGTGGCCGTGTGCTTGTTGAACAAC-(dSpacer)-GGTGACCAATTTGG-(C3 Spacer)

The reverse primer was adapted by adding biotin to the 5′ end. The probe was also modified between 31 bp and 32 bp by incorporating a tetrahydrofuran (THF) site and a fluorescent group at the 5′ end. Additionally, a quenching group was appended to the 3′ end of the probe. Hangzhou Qingke Biotechnology Co. Ltd performed these modifications during primer and probe synthesis.

The common PCR reaction test mixture consisted of a total volume of 20 μL, comprising of 2 μL of 10 × Buffer (Thermo Fisher Scientific), 2 μL of MgCl_2_ (25 mM, Thermo Fisher Scientific), 1.6 μL of dNTPs (25 mM, Thermo Fisher Scientific), 1 μL of forward primer PEFF3 (10 μM), 1 μL of reverse primer PEFR1G (10 μM), 0.4 μL of *Taq* DNA polymerase (5U/μL, Thermo Fisher Scientific), 3 μL of DNA template, and 9 μL of ddH_2_O. The PCR conditions were as follows: initial denaturation at 95 °C for 8 min, followed by 35 cycles of amplification at 94 °C for 40 s, annealing at 62.5 °C for 30 s, and extension at 72 °C for 30 s; the amplification was completed at 72 °C for 10 min. Amplified products were separated by electrophoresis on a 2w% agarose gel (Shanghai Sangon Biotech Co., Ltd.), and the positive PCR product length were 291 bp and subjected to Tsingke Biotechnology Co., Ltd for Sanger sequencing.

The common RPA reaction test was carried out by a commercial Recombinase Polymerase-based Amplification Kit (Haimps Future (Changzhou) Biotechnology Co., Ltd, China). The reaction mixture was 50 μL, containing 2 μL each of the primers (PEFF3 and PEFR1G, 10 μM), 29.4 μL of A Buffer, 5 μL of DNA template, and 9.1 μL of ddH_2_O. In addition, 2.5 μL of B Buffer (magnesium acetate, 21 mM) was added to the solution after 30 min reaction at 37 °C. The product was mixed with an equal amount of 25:24:1 phenol-chloroform (laboratory configuration) to eliminate protein. After centrifugation for 5 min at 12000 rpm, the supernatant was analyzed using electrophoresis on a 2w% agarose gel.

### 2.4 RPA-LFD assay and optimization

The RPA-LFD assay was performed using a commercial Recombinase Polymerase Amplification NFO Kit (Haimps Future (Changzhou) Biotechnology Co., Ltd) combined with LFD (Haimps Future (Changzhou) Biotechnology Co., Ltd). According to the kit instruction, the RPA-LFD reaction system contained 29.4 μL of A Buffer, 1 μL of each RPA primer (PEFF3 and PEFR1G, 10 μM), 0.6 μL of NFO probe (PEFT1, 10 μM), 10.5 μL of ddH_2_O, 5 μL of DNA sample, and 2.5 μL of B Buffer (magnesium acetate, 21 mM). After incubating at 37 °C for 20 min, 10 μL of RPA product was mixed with 90 μL of ddH_2_O and shaken. Following centrifugation at 12000 rpm for 1 min, 50 μL of the diluted product was added to LFD. The results were observed after 3 min. In the following experiments, all LFD visualization reactions were carried out at room temperature and for 3 min, and the later optimizations of this study were only for DNA amplification by RPA reaction except for LFD.

Following conventional RPA-LFD parameters, five temperature gradients (27, 32, 37, 42, and 47 °C) were set to optimize the RPA temperature. The reaction was conducted for 20 min, after which the reaction time gradients of 10, 15, 20, 25 and 30 min were set at the ideal temperature of 37 °C, based on the findings of the reaction temperature screening, to determine the RPA optimal reaction time. These experimental steps were taken out repeatedly three times.

### 2.5 RPA-LFD specificity assay

To evaluate the specificity of the primers-probe set designed in this study, the DNA samples from four nematode species and four fish species were used as controls to test RPA-LFD specificity. The specificity for the common PCR and RPA reactions were compared among these outcomes. The identified *A. simplex (s. s)* and *A. pegreffii* were used as positive controls. Four nematode species, including *Hysterothylacium aduncum, H*. *liparis*, *H*. *sinense,* and *H*. *fabri* were used as negative control. Furthermore, four fish species used as a control included small yellow croaker, hairtail, mackerel and Japanese jack mackerel. Their DNA mixture and ddH_2_O were used as negative samples. These samples were incubated at 37 °C for 20 min for RPA-LFD amplification and specificity assay, and all tests were independently repeated three times.

### 2.6 RPA-LFD sensitivity assay

The *A. simplex (s. s.)* and *A. pegreffii* DNA mixture (concentration ratio 1:1) with an initial concentration of 9.5 ng/μL was used to test the sensitivity, and ddH_2_O was used as a negative control. The DNA mixture was gradually diluted with ddH_2_O in a 10-fold gradient, comprising 7 concentration gradients ranging from 9.5 ng/μL to 9.5 fg/μL. The detection limit of the RPA-LFD assay was determined under the optimal conditions, and the tests were repeated three times independently. The results were then compared with those obtained from PCR and the common RPA reactions.

### 2.7 Validation of the artificially contaminated samples

Five half *A. simplex (s. s.)* and five half *A. pegreffii* with similar size were mixed in a 1:1 ratio and diced, then blended uniformly with 10 g, 25 g, 50 g, 75 g and 150 g of small yellow croaker meat, respectively. Ultrapure water was then added, and the mixture was thoroughly blended to form a homogenous paste. Genomic DNA was extracted from 20 mg surimi using an Animal Tissue Genomic DNA Extraction Kit (Hangzhou Xinjing Biotechnology Co., Ltd.), and then tested using a developed RPA-LFD assay.

## 3 Results

### 3.1 *Anisakis* samples and species identification

This study sampled 54 small yellow croakers and 13 mackerel, and 734 parasites were obtained. All the parasites were preliminarily identified as nematodes. In this sampling survey,the average infection rate was 85.07 % (57/67) in fish samples, and the average intensity of infection was 10.96 parasites per fish. One of the mackerel had the largest number of 63 parasites.

**Fig. 2:**
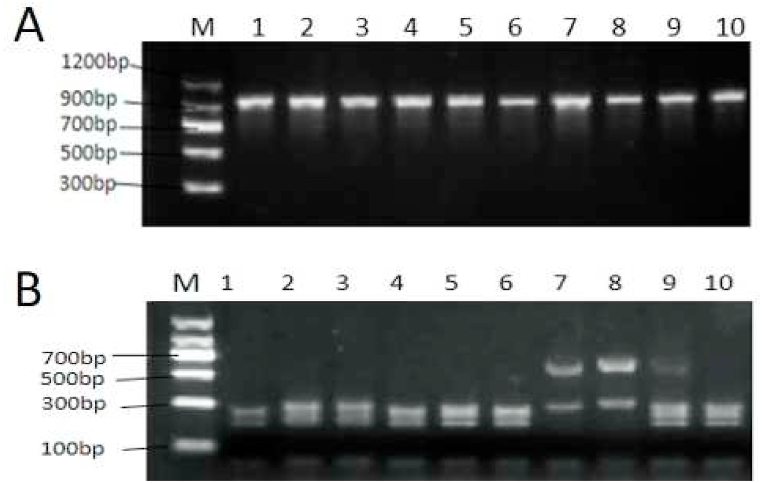
Results of species identification. (A) PCR primers NC5-NC2 amplification results. (B) Result of PCR-RFLP identification. Lanes 1–6 and 10 represent *A. pegreffii*; Lane 7 represents *H. aduncum*; Lane 8 represents *H. fabri*; Lane 9 represents *A. simplex (s. s.)* and *A. pegreffii* hybrid.

The quality of isolated DNA showed that the concentration ranged from 2.5 ng/μL to 26.3 ng/μL, with an average concentration of 7.99 ± 0.13 ng/μL. The purity of A_260_/A_280_ ranged from 1.8 to 2.3 (with an average of 1.94 ± 0.11). The results indicated that there were residual proteins in some of the samples, which could be used for the subsequent analysis of PCR. The primers NC5-NC2 produced a specific lane on agarose gel (Fig. 2A); the fragment length was about 960 bp, which was consistent with expected fragment and the literature (Abattouy et al., 2014). Also, all samples displayed successful and specific amplification. The enzyme digestion patterns indicated that most of the samples belonged to *Anisakis pegreffii*; yet, the NO.7 and 8 samples in Fig. 2B showed significant differences from other samples, suggesting a different species. Consequently, these nematodes (i.e., the NO.7 and 8 samples) were identified as *H*. *aduncum* and *H*. *fabri* by sequencing and Blast in GenBank.

A total of 103 nematodes were tested using PCR-RFLP; 81 were identified to *Anisakis* species and 22 were *Hysterothylacium* species. Among the *Anisakis*, 77 samples were *A. pegreffii* (74.76 %), 1 was *A. simplex (s. s.)* (0.97 %), and 3 were *A. simplex (s. s.)* and *A. pegreffii* hybrid (2.91 %). The 22 *Hysterothylacium* nematodes have been classified into four separate species: 10 samples were *H. aduncum* (9.71 %), 7 were *H. fabri* (6.80 %), 3 were *H. sinense* (2.91 %), and 2 were *H. liparis* (1.95 %). The results indicated that *A. pegreffii* was the most prevalent species, with the following species placed in order of infection: *A. pegreffii* > *H. aduncum* > *H. fabri* > *H. sinense* > *A. simplex (s. s.)* and *A. pegreffii* hybrid > *H. liparis* > *A. simplex (s. s.)*.

### 3.2 Specificity of the RPA-LFD

**Fig. 3:**
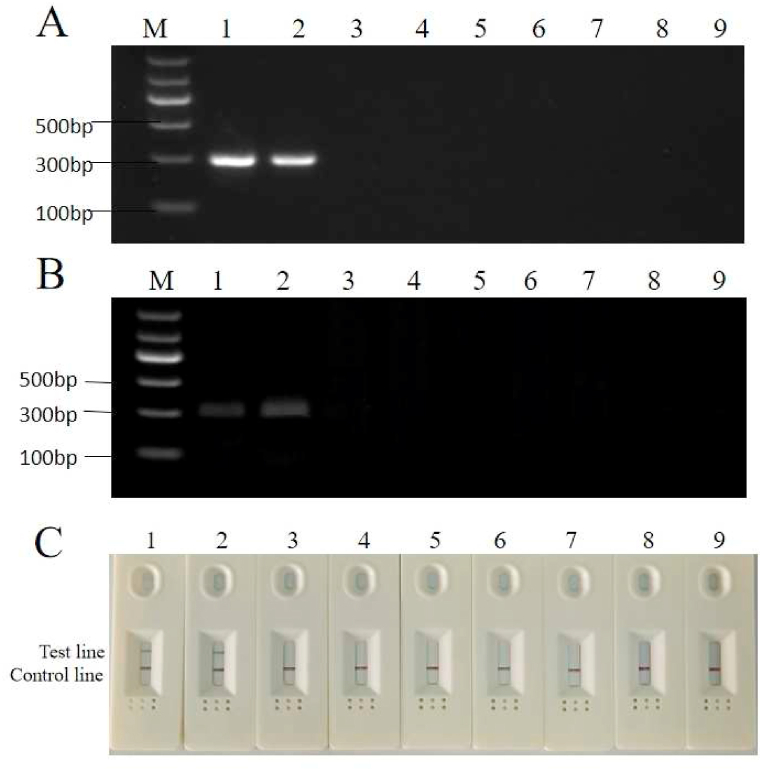
The results for specificity based on PCR (A), RPA (B) and RPA-LFD (C). Lanes 1 represents *A.* simplex (s. s.); Lane 2 represents *A. pegreffii*; Lane 3 represents *H. aduncum*; Lane 4 represents *H. sinense*; Lane 5 represents *H. fabri;* Lane 6 represents *H. liparis*; Lane 7 represents the aggregate sample of four fish species meat; Lane 8 represents small yellow croaker; Lane 9 represents negative control (ddH_2_O was used as a reaction template). (For interpretation of the references to colour in this figure legend, the reader is referred to the Web version of this article.)

Common PCR and RPA tests successfully detected *A. simplex (s. s.)* and *A*. *pegreffii*, as demonstrated in Fig. 3. Both the PCR (Fig. 3A) and common RPA reactions (Fig. 3B) using the designed primers obtained the same electrophoresis results on agarose gel, and the *A. simplex (s. s.)* and *A. pegreffii* DNA also produced clear signal stripes on the detection test line of the LFD in RPA-LFD assay, while the other samples showed negative results (Fig. 3C). This is consistent with the results obtained through PCR and common RPA. These results demonstrated that the primers and probe set have good specificity and no cross-reaction with other species or fish host DNA. Thus, the primers and probe set were proved to be suitable for detecting *A. simplex (s. s.)* and *A. pegreffii* in fish products.

### 3.3 Optimization for temperature and time

**Fig. 4:**
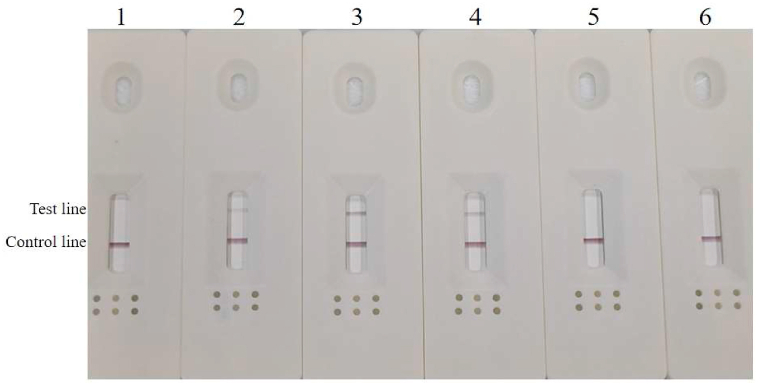
Optimization for RPA-LFD reaction temperature (32–42 °C) for 20 min. Lane 1–5 represent 27 °C, 32 °C, 37 °C, 42 °C, 47 °C; Lane 6 represents negative control (ddH_2_O).

**Fig. 5:**
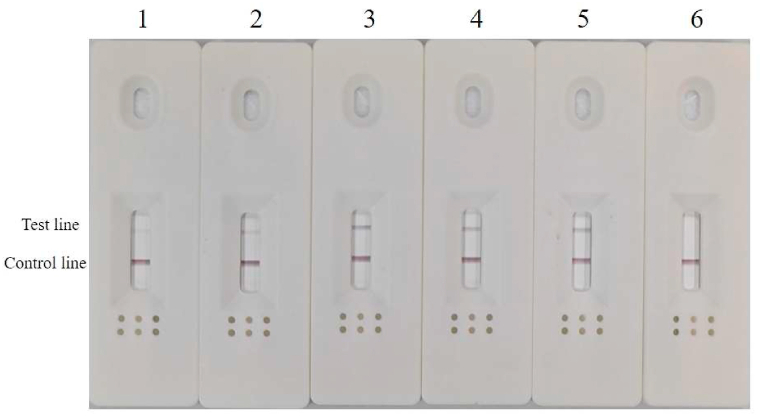
Optimization for RPA-LFD reaction time (10–30 min) at 37 °C. Lane 1–5 represent 10 min, 15 min, 20 min, 25 min, 30 min; Lane 6 represents negative control (ddH_2_O).

RPA-LFD reaction time was set to 20 min based on common conditions, and then an optimization test was conducted to search for the ideal reaction temperature. Fig. 4 shows positive signals observed within the 32–47 °C range, with the most marked signal detected at 37 °C. Consequently, the reaction time was optimized at 37 °C (Fig. 5). Positive products were observed throughout each gradient within 10–30 min, and no noticeable discrepancy was observed between the 20–30 min interval. Therefore, the optimal condition for the developed RPA-LFD was determined to be incubated at 37 °C for 20 min, and the whole process of DNA amplification by RPA and readout by LFD did not exceed 30 min.

### 3.4 RPA-LFD sensitivity assay

**Fig. 6:**
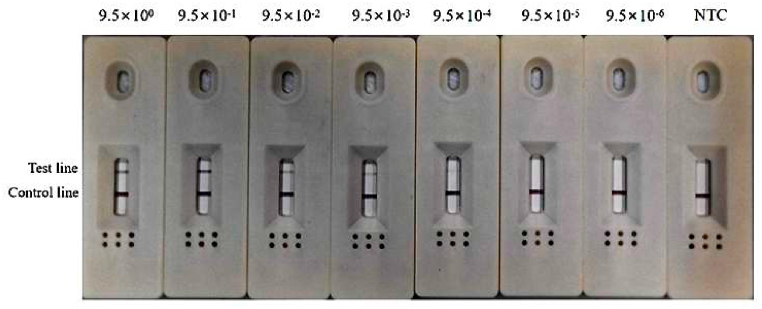
Sensitivity of the RPA-LFD assay. The concentration of DNA template used in this test was 9.5 ng/μL, and all the reaction systems consisted of a 10-fold gradient dilution, with NTC as a ddH_2_O negative control.

**Fig. 7:**
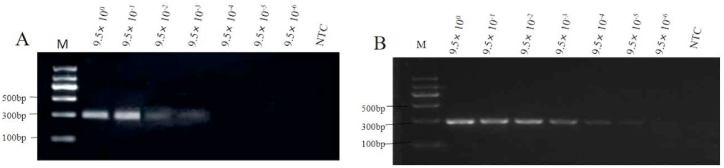
Sensitivity of the common RPA (A) and PCR assays (B) on agarose gel. The concentration of the DNA template used in this test was 9.5 ng/μL, and all the reaction systems consisted of a 10-fold gradient dilution, with NTC as a ddH_2_O negative control.

The positive DNA mixture was used to test the detection limit of RPA-LFD. The signal strength on the test line gradually weakened as the DNA concentration decreased, and a faint test line at the detection was visible at 9.5 × 10^−4^ ng/μL (Fig. 6). The RPA-LFD detection sensitivity was higher than the common RPA agarose gel separation with a limit of 9.5 × 10^−3^ ng/μL (Fig. 7A) but lower than that of standard PCR agarose gel separation with a limit of 9.5 × 10^−5^ ng/μL (Fig. 7B). This sensitivity is similar with the other report (6 × 10^−4^ ng/μL) [31], and fully meet the requirements for food safety rapid screening.

### 3.5 Validation of the artificially contaminated samples

**Fig. 8:**
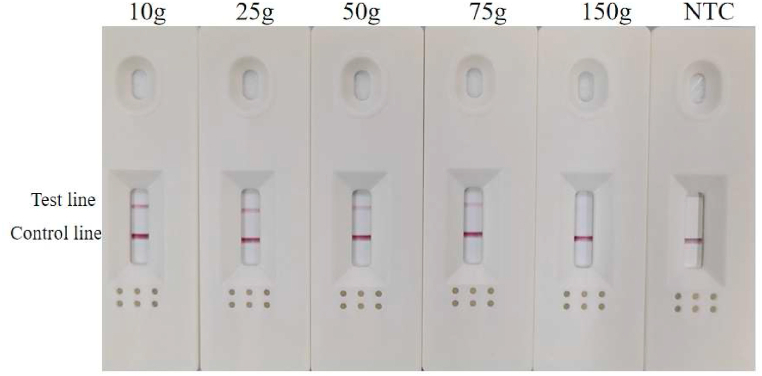
RPA-LFD test results for artificial contaminated samples.

To assess the practicality of developed RPA-LFD for detecting *A. simplex (s. s.)* and *A. pegreffii* contaminated processed fish meat and products, the detectability of this method was assessed using deliberately contaminating fabricated samples. The test procedure was carried out according to the description above. The validation results showed that the test line of all the contaminated samples ranging from 10 g to 75 g indicated positive signals (Fig. 8), while the test line signal remained faintly visible in the mixed fish meat sample contaminated with 150 g. According to the simulated result, one nematode weight approximately 1 mg [26], the results showed that RPA-LFD assay can be used to detect *Anisakis* in surimi fish products, and meant that the method was capable of detecting one larva of 1 mg in 75 g of fish meat (13.3 ppm).

## 4 Discussion

Eating fish and seafood is very important for its proven health benefits and positive impact on the environment. Based on a comparison of 165 countries in 2021, China ranks the highest in fish consumption, followed by Indonesia and India (Naylor et al., 2021). Yet, aquatic food products are not without risk from parasitic zoonoses. In the present study, the prevalence of *Anisakis* infection was 85.03 % (Fig. S2) in small yellow croakers and mackerel from the East China Sea. The main identified nematodes included *A. pegreffii*, *H. aduncum*, and *A. simplex (s. s.)*. Compared to the recent reports (Cai et al., 2023; Li et al., 2023; Lin et al., 2023; Zheng et al., 2023), the quantity ranking of the PCR-RFLP sampling identification results indicated that the frequencies of infections were different, but these results were enough to prove the existence of these parasites in the East China Sea and the high infection rate in marine fish. The number of *A. simplex (s. s.)* was lower than other reports in this sea area, which could be attributed to the different sampling methods, timing, and individual host variations. *A. pegreffii* and *A. simplex (s. s.)* were the main, persistent species in this area (Du et al., 2014; Lin et al., 2023) and the main pathogen of anisakiasis (Chen & Zhang; Uña-Gorospe et al., 2018). Thus, monitoring and detecting *Anisakis* are crucial for ensuring marine fish safety.

The emergence and improvement of modern animal species identification techniques, such as PCR, real-time PCR (qPCR), and enzyme-linked immunosorbent assay (ELISA), have helped to identify *Anisakis*; however, these methods have certain limitations. For example, PCR-based and real-time PCR methods require trained professionals and sophisticated temperature equipment; LAMP assay requires heating the system to 65 °C with a heater (Cammilleri et al., 2020), which restricts its application in economically underdeveloped areas and certain poorly equipped laboratories. Moreover, a longer reaction time (35min) is required for a LAMP assay, and approximately 40 cycles (40 min) are required in the qPCR assay [26,29,30,32]. On the other hand, PCR-RFLP assay demands a specific endonuclease and takes 2.5–4 h [45,46], making these methods unfeasible for rapid on-site detection. Thus, it is imperative to develop a rapid, simple and dependable method to improve the detection effectiveness of *Anisakis*, ensuring precision.

The RPA-LFD assay is a detection technique that combines recombinase polymerase amplification and visualization techniques, showing much strength in pathogen detection. The ITS sequences have high interspecies variability in *Anisakis* and are often used as target genes [47]. In this study, we focused on specific ITS sequences and used them as the detection target to develop an RPA-LFD assay. The specific primers and probe were designed to accomplish this method, and the optimal conditions were screened and refined. The results showed that the RPA-LFD reaction system achieves a successful objective within a temperature range of 32°C–42 °C for 10–20 min, and the whole process should not exceed 30 min, with a detection sensitivity at 9.5 × 10^−4^ ng/μL, and the detection limit in terms of concentration was 13.3 ppm that can detect a single larva in 75 g of marine fish tissue. The limit of detection (LOD) of RPA-LFD assay proposed was 3 times lower than the real-time PCR method [26]. On the other hand, the assay can be carried out at room temperature and has no requirement for expensive and bulky equipment; it is used in conjunction with a direct PCR kit and is appropriate for on-site testing. In addition, compared to common LAMP and PCR-based assays, this method has many advantages, such as room temperature demands, effortless primer design, visualization result with no need for reaction product handling and heightened specificity. Chen et al. [48] reported the common RPA assay for identifying *A. simplex (s. s.)*. Compared to the reported method, this RPA-LFD assay has specificity advantages, i.e., prime higher specificity and probe specificity. As a result, the combination of RPA with the LFD simplifies the test process and yields simpler visualization of results, making rapid on-site detection feasible. Compared to the combination of RPA with SYBR Green detection for *Anisakis* reported by Chen et al. [47] for on-site detection, this study was simpler regarding the visualization results because of using the LFD. In addition, visualization results avoid the harm from UV light using SYBR Green and reduce the error possibility from naked-eye observation. Surprisingly, the sensitivity of RPA-LFD was lower than that of the common PCR agarose gel assay when using the same primers. This result is inconsistent with the previously reported results [49,50]. This difference may have resulted from the primer specificity under room temperature being lower than that of PCR, resulting in non-specific amplification and primer dimer. This disadvantage can be made up by optimizing the conditions for RPA to minimize false-positive results, as suggested in other reports [41,43]. Furthermore, the quality of DNA extracted methods may also affect the results of the RPA-LFD assay, and the high quality and concentration of pure DNA can improve its sensitivity and efficiency. Additionally, the LFD signal only reveals the existence of specific substances in the RPA product and determines the rough concentration based on the LFD signal strength [37]. This approach fails to meet quantitative requirements for food testing and the prospect of high-throughput detection. In the future, with the high-throughput detection technology demand, this assay can be improved through the combination of multiple LFD or multiple RPA-LFD assays [51] because this is an important trend and direction in the field of detection technology.

## 5 Conclusion

In this study, an infect rate investigation and molecular identification of *Anisakis* in small yellow croaker and mackerel in the East China Sea was conducted, and a visual detection assay was developed based on RPA-LFD. This new rapid (completed within 30 min) and efficient method can detect *A.simplex (s. s.)* and *A. pegreffii* in seafood with high specificity and sensitivity. Additionally, it does not rely on instrumentation or complex operation, providing a technological solution for simple and rapid detection of *A. simplex (s. s.)* and *A. pegreffii* in fish meat and processed products.

## Ethical approval

All animal procedures were performed according to the guidelines of the China Council on Animal Care, and the protocols were approved by the Experimental Animal Management Committee of China Jiliang University (approval number: 2024–014). Informed consent was not required for this study because this article does not involve any patient information.

## Data availability

The data has not been uploaded to a publicly available repository, and the data that support the findings of this study are available on request from the corresponding author, Guan, upon reasonable request.

## CRediT authorship contribution statement

**Xiaoming Wang:** Writing – review & editing, Writing – original draft, Visualization, Validation, Methodology, Data curation, Conceptualization. **Ting Xu:** Investigation, Data curation. **Siling Ding:** Validation, Data curation. **Ye Xu:** Validation, Formal analysis. **Xingsheng Jin:** Writing – review & editing, Supervision, Resources. **Feng Guan:** Writing – review & editing, Supervision, Project administration, Methodology, Conceptualization.

## Declaration of competing interest

The authors declare that they have no known competing financial interests or personal relationships that could have appeared to influence the work reported in this paper.
