# Supplementary material for: Recombinase polymerase amplification combined with lateral flow dipstick assay for rapid visual detection of A.simplex (s. s.) and A.pegreffii in sea foods
Source: Heliyon. 2024 Apr 7;10(7):e28943. doi: 10.1016/j.heliyon.2024.e28943 (PMC11016599; doi:10.1016/j.heliyon.2024.e28943)
Supplement: Multimedia component 1 [file mmc1.docx]

Fish samples are sourced from Zhoushan Island, Zhejiang Province, China, and their positions shown in **Fig. S1.** It is worth mentioning that fish samples from locations A, B, and C were obtained through capture, while samples from location D were purchased from local fish markets.


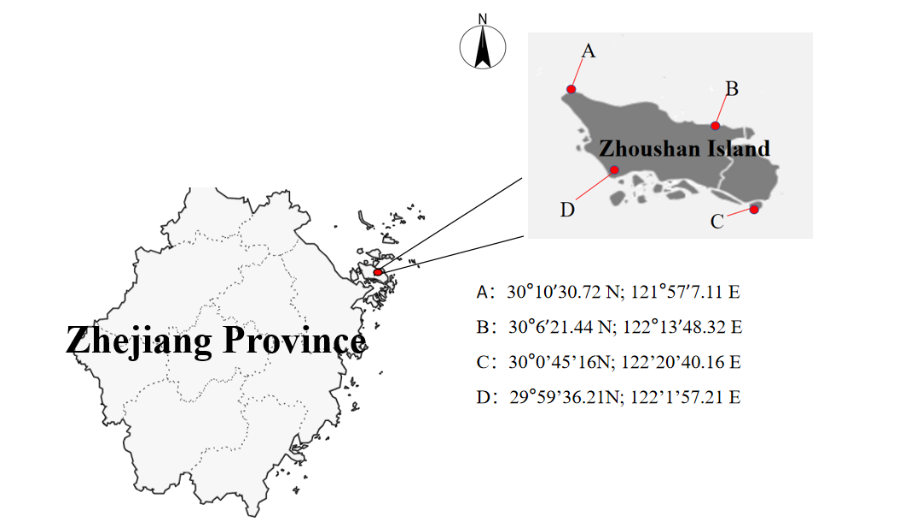


**Fig. S1** Fish sample capture location.

The Fish sample dates are listed in **Table S1**, and the statistics listed in **Fig.S2**

**Table S1**

Fish samples data and infection status. Recorded with their total body length, weight, gender and the total number of Anisakis spp.

| **Sample Number** | **Sample Species Name** | **Sample Gender** | **Sample Weight**  **（g）** | **Sample Length（cm）** | **Number Of Infections** | **Sampling**  **Location** |
| --- | --- | --- | --- | --- | --- | --- |
| 1 | small yellow croaker | female | 33.09 | 16.0 | 12 | A |
| 2 | small yellow croaker | male | 27.57 | 16.1 | 1 | A |
| 3 | small yellow croaker | female | 34.62 | 15.7 | 12 | A |
| 4 | small yellow croaker | female | 50.10 | 17.6 | 14 | A |
| 5 | small yellow croaker | female | 49.87 | 18.1 | 15 | A |
| 6 | small yellow croaker | male | 34.74 | 16.5 | 28 | A |
| 7 | small yellow croaker | female | 33.93 | 16.3 | 12 | A |
| 8 | small yellow croaker | female | 30.84 | 16.5 | 16 | A |
| 9 | small yellow croaker | female | 35.25 | 17.85 | 16 | A |
| 10 | small yellow croaker | female | 36.58 | 17.3 | 23 | A |
| 11 | small yellow croaker | female | 27.41 | 16.2 | 18 | A |
| 12 | small yellow croaker | male | 35.29 | 19.0 | 11 | A |
| 13 | small yellow croaker | female | 33.35 | 16.5 | 19 | A |
| 14 | small yellow croaker | male | 38.96 | 18.5 | 15 | A |
| 15 | small yellow croaker | female | 27.39 | 15 | 17 | A |
| 16 | small yellow croaker | female | 41.95 | 17.5 | 28 | A |
| 17 | small yellow croaker | female | 35.12 | 16.4 | 11 | A |
| 18 | small yellow croaker | female | 38.23 | 17.2 | 20 | A |
| 19 | small yellow croaker | male | 33.34 | 17.3 | 19 | A |
| 20 | small yellow croaker | female | 42.69 | 18.7 | 20 | A |
| 21 | small yellow croaker | female | 29.86 | 15.4 | 16 | A |
| 22 | small yellow croaker | female | 40.09 | 18.5 | 12 | A |
| 23 | small yellow croaker | female | 48.27 | 19.5 | 32 | A |
| 24 | small yellow croaker | male | 29.12 | 15.9 | 4 | B |
| 25 | small yellow croaker | female | 44.67 | 17.0 | 16 | B |
| 26 | small yellow croaker | male | 25.21 | 15.9 | 19 | B |
| 27 | small yellow croaker | female | 28.22 | 16.2 | 49 | B |
| 28 | small yellow croaker | female | 24.37 | 16.1 | 12 | B |
| 29 | small yellow croaker | female | 32.57 | 16.1 | 30 | B |
| 30 | small yellow croaker | female | 32.48 | 17.2 | 4 | B |
| 31 | small yellow croaker | male | 34.36 | 14.2 | 36 | B |
| 32 | small yellow croaker | male | 25.20 | 15.5 | 27 | B |
| 33 | small yellow croaker | female | 33.00 | 15.4 | 17 | B |
| 34 | small yellow croaker | male | 30.81 | 16.5 | 14 | B |
| 35 | mackerel | male | 60.13 | 17.49 | 3 | C |
| 36 | mackerel | female | 67.64 | 19.23 | 1 | C |
| 37 | mackerel | female | 56.98 | 18.22 | 1 | C |
| 38 | mackerel | male | 57.32 | 17.51 | 3 | C |
| 39 | mackerel | female | 66.70 | 17.79 | 3 | C |
| 40 | mackerel | female | 68.00 | 19.67 | 2 | C |
| 41 | mackerel | female | 71.96 | 18.56 | 1 | C |
| 42 | mackerel | female | 65.41 | 19.51 | 1 | C |
| 43 | mackerel | male | 100.76 | 22.10 | 7 | C |
| 44 | mackerel | male | 103.91 | 19.70 | 2 | C |
| 45 | mackerel | male | 92.47 | 18.90 | 0 | C |
| 46 | mackerel | male | 112.70 | 20.33 | 3 | C |
| 47 | mackerel | female | 1372.62 | 78.23 | 63 | C |
| 48 | small yellow croaker | female | 43.43 | 17.78 | 4 | D |
| 49 | small yellow croaker | male | 35.05 | 16.82 | 0 | D |
| 50 | small yellow croaker | male | 32.91 | 16.30 | 1 | D |
| 51 | small yellow croaker | male | 35.24 | 16.42 | 5 | D |
| 52 | small yellow croaker | male | 37.62 | 17.92 | 4 | D |
| 53 | small yellow croaker | male | 34.41 | 16.56 | 2 | D |
| 54 | small yellow croaker | female | 38.87 | 16.75 | 1 | D |
| 55 | small yellow croaker | female | 34.78 | 16.81 | 1 | D |
| 56 | small yellow croaker | female | 31.71 | 16.43 | 0 | D |
| 57 | small yellow croaker | female | 34.04 | 16.85 | 3 | D |
| 58 | small yellow croaker | female | 42.93 | 18.80 | 0 | D |
| 59 | small yellow croaker | female | 33.80 | 16.44 | 0 | D |
| 60 | small yellow croaker | male | 38.95 | 18.11 | 1 | D |
| 61 | small yellow croaker | female | 45.42 | 17.03 | 4 | D |
| 62 | small yellow croaker | male | 27.12 | 15.20 | 0 | D |
| 63 | small yellow croaker | female | 33.93 | 16.30 | 0 | D |
| 64 | small yellow croaker | male | 31.18 | 15.60 | 0 | D |
| 65 | small yellow croaker | male | 33.79 | 16.66 | 3 | D |
| 66 | small yellow croaker | male | 32.61 | 16.49 | 0 | D |
| 67 | small yellow croaker | male | 35.04 | 17.33 | 0 | D |


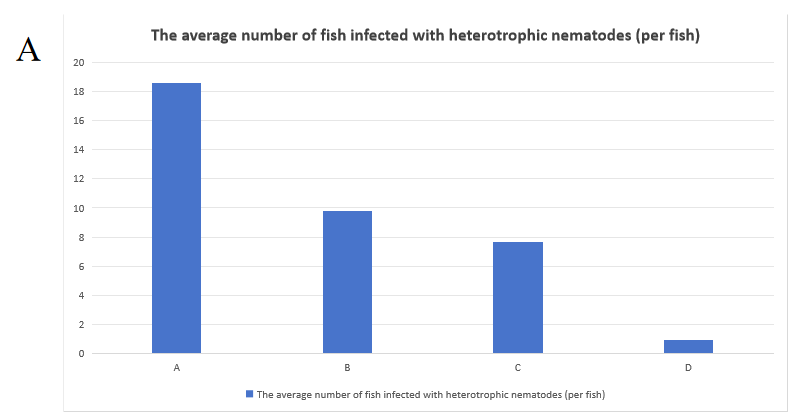

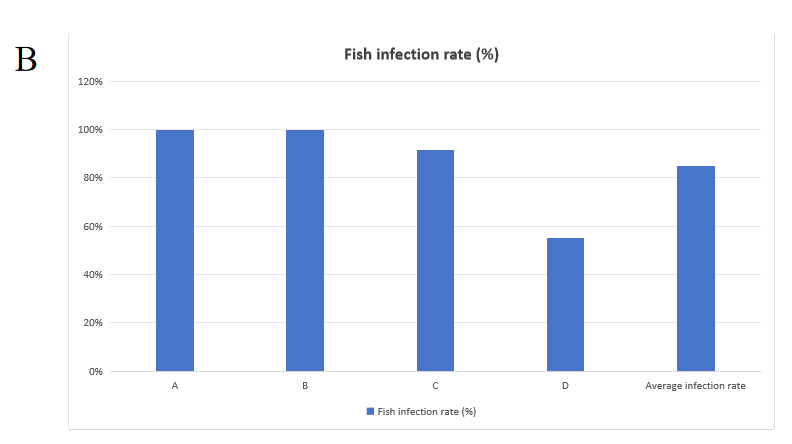


**Fig. S2.** Statistics on fish infection status data.
